# P-1079. *In Vitro* and *in Vivo* Antiviral Activity of S-892216, a Second-Generation Oral 3CL^pro^ Inhibitor against SARS-CoV-2

**DOI:** 10.1093/ofid/ofae631.1267

**Published:** 2025-01-29

**Authors:** Haruaki Nobori, Sho Kawashima, Reiko Dodo, Yuki Maruyama, Takayo Haruna, Keiichiro Hirai, Yuto Unoh, Kenji Nakahara, Shota Uehara, ryosuke watari, tomoyuki kawachi, yuka Natsume, katsumoto hata, yukiko orita, kae fujisawa, tetsuya miyano, hideko kaneda, taeko kawai, Kae Inoue, Akane Hayashi, Takashi Hashimoto, Kazumi Matsumoto, Kaoru Baba, taichi ueda, takafumi sato, Teruhisa Kato, Keita Fukao

**Affiliations:** SHIONOGI & CO., LTD., Toyonaka-shi, Osaka, Japan; Shionogi & Co., Ltd., Toyonaka, Osaka, Japan; Shionogi & Co., Ltd., Toyonaka, Osaka, Japan; Shionogi & Co., Ltd., Toyonaka, Osaka, Japan; Shionogi & Co., Ltd., Toyonaka, Osaka, Japan; Shionogi & Co., Ltd., Toyonaka, Osaka, Japan; Shionogi & Co., Ltd., Toyonaka, Osaka, Japan; Shionogi & Co., Ltd., Toyonaka, Osaka, Japan; SHIONOGI & CO., LTD., Toyonaka-shi, Osaka, Japan; Shionogi & Co., Ltd., Toyonaka, Osaka, Japan; Shionogi & Co., Ltd., Toyonaka, Osaka, Japan; Shionogi & Co., Ltd., Toyonaka, Osaka, Japan; Shionogi & Co., Ltd., Toyonaka, Osaka, Japan; Shionogi & Co., Ltd., Toyonaka, Osaka, Japan; Shionogi & Co., Ltd., Toyonaka, Osaka, Japan; Shionogi & Co., Ltd., Toyonaka, Osaka, Japan; Shionogi TechnoAdvance Research Co., Ltd., Toyonaka, Osaka, Japan; Shionogi TechnoAdvance Research Co., Ltd., Toyonaka, Osaka, Japan; Shionogi TechnoAdvance Research Co., Ltd., Toyonaka, Osaka, Japan; Shionogi TechnoAdvance Research, Co., Ltd., Toyonaka, Osaka, Japan; Shionogi TechnoAdvance Research Co., Ltd., Toyonaka, Osaka, Japan; Shionogi TechnoAdvance Research, Co., Ltd., Toyonaka, Osaka, Japan; Shionogi TechnoAdvance Research, Co., Ltd., Toyonaka, Osaka, Japan; Shionogi & Co., Ltd., Toyonaka, Osaka, Japan; Shionogi & Co., Ltd., Toyonaka, Osaka, Japan; Shionogi & Co., Ltd., Toyonaka, Osaka, Japan; Shionogi&Co., Ltd., Toyonaka, Osaka, Japan

## Abstract

**Background:**

COVID-19 caused by SARS-CoV-2 remains a global public health concern. Although oral direct-acting antivirals for COVID-19 (such as molnupiravir, nirmatrelvir/ritonavir, ensitrelvir) were approved for clinical use, there are concerns about drug-drug interactions (DDI) and safety, so development of new therapeutics is needed. In this study, we describe enzyme inhibitory and antiviral activity of S-892216, a second-generation small molecular 3C-like protease (3CL^pro^) inhibitor.

**Methods:**

The 3CL^pro^ enzymatic assay was conducted by mass spectrometry system. *In vitro* antiviral activity was evaluated using VeroE6/TMPRSS2 cells and human airway epithelial cells (hAEC) following infection by several SARS-CoV-2 variants. *In vivo* efficacy was evaluated using Balb/c mice, intranasally infected with SARS-CoV-2, and S-892216 was orally administered.

**Results:**

S-892216 showed high 3CL^pro^ inhibitory activity (IC_50_ = 0.697 nmol/L) and exhibited *in vitro* antiviral activity against several SARS-CoV-2 strains, including Omicron variants (EC_50_ = 2.27-12.5 nmol/L in VeroE6/TMPRSS2 cells, EC_90_ = 2.31-2.41 nmol/L in hAECs). Furthermore, S-892216 suppressed lung virus titer in Balb/c mice infected with SARS-CoV-2 in a dose-dependent manner.

**Conclusion:**

S-892216 has stronger 3CL^pro^ inhibitory and antiviral activity than approved 3CL^pro^ inhibitors and has been confirmed to be effective *in vivo*. Due to the strong antiviral activity of S-892216, it is suggested to be effective at low doses in clinical settings. DDI and safety will be evaluated in clinical trials.

**Disclosures:**

**Haruaki Nobori, PhD**, Shionogi & Co., Ltd.: Employee|Shionogi & Co., Ltd.: Stocks/Bonds (Private Company) **Sho Kawashima**, Shionogi & Co., Ltd.: Employee **Reiko Dodo, n/a**, Shionogi & Co., Ltd.: Employee **Yuki Maruyama, PhD**, Shionogi & Co., Ltd.: Employee|Shionogi & Co., Ltd.: Stocks/Bonds (Private Company) **Takayo Haruna, n/a**, Shionogi & Co., Ltd.: Employee **Keiichiro Hirai, PhD**, Shionogi & Co., Ltd.: Employee **Yuto Unoh, PhD**, Shionogi & Co., Ltd.: Employee **Kenji Nakahara, PhD**, Shionogi & Co., Ltd.: Employee **Shota Uehara, Ph.D.**, Shionogi & Co., Ltd.: Employee|Shionogi & Co., Ltd.: Stocks/Bonds (Private Company) **ryosuke watari, n/a**, Shionogi & Co., Ltd.: Employee **tomoyuki kawachi, n/a**, Shionogi & Co., Ltd.: Employee **yuka Natsume, n/a**, Shionogi & Co., Ltd.: Employee **katsumoto hata, n/a**, Shionogi & Co., Ltd.: Employee **yukiko orita, n/a**, Shionogi & Co., Ltd.: Employee **kae fujisawa, n/a**, Shionogi & Co., Ltd.: Employee **tetsuya miyano, PhD**, Shionogi & Co., Ltd.: Employee **hideko kaneda, n/a**, Shionogi TechnoAdvance Research Co., Ltd.: Employee **taeko kawai, n/a**, Shionogi TechnoAdvance Research Co., Ltd.: Employee **Kae Inoue, n/a**, Shionogi & Co., Ltd.: Stocks/Bonds (Private Company)|Shionogi TechnoAdvance Research CO., Ltd.: employee **Akane Hayashi, n/a**, Shionogi & Co., Ltd.: Stocks/Bonds (Private Company)|Shionogi TechnoAdvance Research: Employee **Takashi Hashimoto, n/a**, Shionogi TechnoAdvance Research CO., Ltd.: employee **Kazumi Matsumoto, n/a**, Shionogi TechnoAdvance Research: Employee **Kaoru Baba, n/a**, Shionogi TechnoAdvance Research: Employee **taichi ueda, PhD**, Shionogi & Co., Ltd.: Employee **takafumi sato, PhD**, Shionogi & Co., Ltd.: Employee|Shionogi & Co., Ltd.: Stocks/Bonds (Private Company) **Teruhisa Kato, PhD**, Shionogi & Co., Ltd.: employee|Shionogi & Co., Ltd.: Stocks/Bonds (Private Company) **Keita Fukao, MD**, SHIONOGI and CO., LTD.: Stocks/Bonds (Private Company)

